# Integrated transcriptomic and metabolomic profiling identifies *IbADCL1* as a key regulator of folate biosynthesis in sweet potato storage roots

**DOI:** 10.1016/j.fochms.2025.100302

**Published:** 2025-09-13

**Authors:** Qingming Ren, Yingzhi Wu, Huiyu Gao, Qi Ma, Xinli Liu, Yinghui Li, Xiaoxi Zhen, Yuanhuai Han, Bin Zhang

**Affiliations:** a College of Agriculture, Shanxi Agricultural University, Taigu 030801, Shanxi, China; b Houji Laboratory in Shanxi Province, Taiyuan 030031, Shanxi, China

**Keywords:** Metabolomics, Transcriptomics, Biofortification, Vitamin B9, Nutritional enhancement

## Abstract

We hypothesized that key regulatory genes in the folate biosynthesis pathway could be identified through integrated multi-omics analysis and functionally validated to enhance folate accumulation in sweet potato storage roots. Folate, an essential micronutrient in plant metabolism and human diets, shows poorly characterized accumulation mechanisms in sweet potato storage roots. Comparative profiling of 26 cultivars identified low- (968–19) and high-folate (Y25) varieties. Integrated multi-omics analysis of tuber tissues across developmental stages revealed 5-methyltetrahydrofolate (5-MTHF) and 5-formyltetrahydrofolate (5-FTHF) as principal determinants of folate variation. Differential expression analysis pinpointed *IbADCL1*, encoding 4-amino-4-deoxychorismate lyase, as a putative regulatory gene. Heterologous overexpression of *IbADCL1* in 968–19 triggered 177–222% increases in total folate, with 5-MTHF and 5-FTHF levels elevated by 184–224% and 40–142%, respectively, versus wild-type controls. This study establishes *IbADCL1* as a rate-limiting controller of folate biosynthesis, offering molecular targets for metabolic engineering to enhance nutritional quality in sweet potato*.*

## Highlights

• Discovery of IbADCL1 as a key folate regulator in sweet potato storage roots.
• Multi-omics identified 5-MTHF and 5-FTHF as major folate forms in sweet potato.
• Overexpression of *IbADCL1* boosted total folate by 177–222% in low-folate lines.
• Developmental folate peaks aligned with dry matter accumulation stages (S3–S4).

## Graphical abstract

**Figure:**
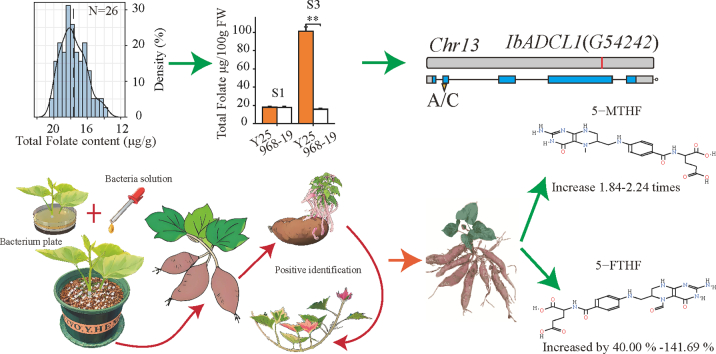

## 1 Introduction

Sweet potato (*Ipomoea batatas* (L.) Lam.), a globally vital staple crop, is renowned for its nutritional richness, including carotenoids, anthocyanins, and polysaccharides (Zong et al., 2023). Despite its agricultural significance, folate (vitamin B9) content in sweet potato remains suboptimal, with limited understanding of its biosynthetic regulation (Low & Thiele, 2020). Folate deficiency, affecting over 20% of the global population, is linked to severe health disorders, underscoring the urgency of biofortification efforts in staple crops such as sweet potato (Gutierrez-Valdes et al., 2020).

As humans cannot synthesize folate and must obtain it from dietary sources (Nawaz & Kipreos, 2022; Palchetti et al., 2020), maintaining adequate levels is critical for physiological functions including nucleic acid synthesis and methylation reactions, primarily mediated by its active form 5-methyltetrahydrofolate (5-MTHF). The recommended daily intake is 400 μg for adults and 600 μg during pregnancy (Chen et al., 2019; de Crécy-Lagard, El Yacoubi, de la Garza, Noiriel, & Hanson, 2007), yet natural folate in plants is often low and easily degraded during food processing and cooking (Blancquaert et al., 2010; de Crécy-Lagard et al., 2007). Studies have shown that excessive consumption of tea or coffee (Sijilmassi, 2019), alcohol abuse (Badian & Dzierżanowski, 2018), and smoking (Sijilmassi, 2019) may negatively affect the utilization, absorption, and storage of folate in the body, thereby disrupting key metabolic processes and impairing cell growth and development (Banyś, Knopczyk, & Bobrowska-Korczak, 2020). Folate plays a crucial role in preventing neural tube defects (Asbaghi et al., 2021), reducing the risk of megaloblastic anemia (Badian & Dzierżanowski, 2018), maintaining normal neurological function (Asbaghi et al., 2021), and lowering the risk of certain cancers (Hossain et al., 2004). These limitations of conventional dietary interventions highlight the need for biotechnology-based biofortification to enhance folate content and stability in food crops.

The folate biosynthesis pathway is well-characterized in model plants, requiring coordinated activity between mitochondria (pterin synthesis) and plastids (p-aminobenzoate (PABA) synthesis), culminating in cytoplasm where tetrahydrofolate (THF) is formed by 4-amino-4-deoxychorismate lyase (ADCL) (Storozhenko et al., 2007). Post-translational regulation via polyglutamylation further modulates folate stability and trafficking (Hanson, Lin, Carmo-Silva, & Parry, 2016). However, these mechanisms are conserved, their spatiotemporal regulation in storage organs of polyploid crops like sweet potato remains largely unknown.

Emerging evidence establishes genetic and metabolic engineering as pivotal strategies for folate biofortification in crops. Targeted manipulation of folate biosynthesis genes in *Arabidopsis thaliana* revealed that knockout of folylpolyglutamate synthase gene (*FPGS*) or overexpression of gamma-glutamyl hydrolase gene (*GGH*) reduced total folate content by 40–45%, while suppression of GGH enzymatic activity enhanced folate accumulation by 30% (Hou et al., 2022). Parallel efforts in rice (*Oryza sativa*) demonstrated 8–17% and 1.2–21.2% folate increases through overexpression of dihydrofolate reductase gene (*DHFS*) and *FPGS*, respectively (Orsomando et al., 2005). Functional validation in maize (*Zea mays*) identified a hyperactive *ZmADCL2* mutant (*Mut9264*) with a 4-bp exon insertion, elevating kernel folate levels 1.37-fold versus wild-type controls (Jiao et al., 2025). Notably, heterologous expression of *SiADCL1* in *Arabidopsis* boosted folate content 1.14–1.84-fold but induced delayed flowering, suggesting pleiotropic regulation (Zhao et al., 2024). These findings collectively confirm that precise modulation of folate pathway genes enhances crop folate content, positioning genetic engineering as a transformative tool for nutritional improvement in staple crops.

The hexaploid genome architecture (2n = 6× = 90) and inherent heterozygosity of sweet potato present significant challenges for conventional breeding strategies (Yan et al., 2022). Compounding these genetic complexities, viral pathogenesis has emerged as a devastating constraint on crop improvement (Wang, Poque, & Valkonen, 2019). Recent advances in Agrobacterium-mediated transformation systems, particularly utilizing rhizogenic (Ri) and tumorigenic (Ti) plasmid vectors, offer promising solutions for genetic manipulation in dicotyledonous species (Gutierrez-Valdes et al., 2020; Zhong et al., 2024). Notable progress in hairy root transformation has been achieved across various horticultural crops through systematic protocol optimization. In *Fragaria × ananassa*, *A. rhizogenes*-mediated transformation of cotyledon explants yielded highly fluorescent root systems with functional metabolic activity (Yan et al., 2023). Citrus transformation studies demonstrated that parameter optimization can enhance transformation efficiency to 96.1% while reducing procedural duration by 40% (Wang, Qin, Wei, Xue, & Dai, 2023). Functional validation of anthocyanin regulatory genes in *Litchi chinensis* was accomplished through stem segment transformation, establishing an efficient platform for secondary metabolite research (Qin et al., 2021). The recent telomere-to-telomere (T2T) genome assembly of hexaploid sweet potato (Xiao et al., 2025) and the development of tissue culture-independent Cut-Dip-Budding (CDB) transformation (Cao et al., 2023) now enable precise genetic manipulation in this crop. Critical advancements in *I. batatas* transformation include the successful regeneration of five transgenic cultivars via *A. rhizogenes*-mediated protocols (Yu et al., 2020). These developments provide essential molecular tools for overcoming genetic constraints in species, thereby enabling targeted trait modification and advancing functional genomics research.

However, the critical regulatory nodes governing folate accumulation in sweet potato storage roots remain undefined. Given this knowledge gap, we hypothesized that key biosynthetic genes exhibit stage-specific expression patterns that correlate with folate content. To test this, we employed a multi-omics approach integrating metabolomic and transcriptomic analyses of contrasting germplasm across different developmental stages to identify critical regulatory gene candidates. Furthermore, leveraging recent advancements in transformation technology, we aimed to functionally validate the role of the prime candidate through metabolic engineering in a low-folate cultivar. This study provides mechanistic insight into folate homeostasis in sweet potato and demonstrates a practical strategy for its biofortification.

## 2 Materials and methods

### 2.1 Plant materials and growth conditions

Total folate content was systematically quantified in storage roots of 26 sweet potato resources (Supplementary Data Table S1) during root expansion phase in 2021 to screen representative varieties with significant folate differences. Extreme genotypes Y25 (“Yanshu 25”, high folate) and 968–19 (“Shangshu 19”, low folate) were selected for further analysis. These selected varieties were subsequently cultivated in pot culture at the greenhouse of Coarse Cereal Molecular Breeding Team, Shanxi Agricultural University in mid-May 2022. Plants were grown under controlled conditions: 14-h photoperiod (600 μmol/m^2^/s), 25/18 °C day/night. Soil composition was 60% peat, 30% vermiculite, and 10% perlite (pH 6.5). The experimental design included three biological replicates with consistent soil fertility and light conditions maintained across all groups. Nine pots were allocated per variety with one plant per pot. Sampling of smooth-surfaced tubers occurred at 90 days (S1), 120 days (S2), 150 days (S3), and 180 days (S4) post-planting. In a clean workbench environment, the skin of the tubers was peeled off, and the peeled tubers were cut into 0.5 cm^3^ pieces, which were then quickly transferred to 50 mL centrifuge tubes. Subsequently, the samples were immediately immersed in liquid nitrogen and stored at −80 °C for future experimental use.

### 2.2 Determination and analysis of total folate content

In this study, 5 g of visually intact sweet potato storage roots were selected and placed in a 50 mL centrifuge tube, where they underwent freeze-drying for 48 h using a freeze dryer (Marin Christ, Osterode, Germany). The freeze-dried sweet potato roots were then ground into a powder. Subsequently, 0.1 g of the sweet potato powder was accurately aliquoted into a 2 mL centrifuge tube, and 900 μL of phosphate-buffered saline (pH = 7.0) was added. Folate/Vitamin B9 (FA/VB9) enzyme-linked immunosorbent assay (ELISA) kits (Beijing Winter Song Boye Biotechnology Co., Beijing, China) were used to measure the absorbance (OD value) at λ = 450 nm. The folate content in each sample was calculated using the standard curve equation y = 0.0025*×* + 0.019 (R^2^ = 0.9913). The folate content (X) in the sample was calculated using the following formula:

$$X=\left[\left(C-P\right)\times V_{1}\times F/m\times V_{2}\right]\times \left(1000/100\right).$$

*C* represents the OD value of the sample, *P* represents the OD value of the blank, V_1_ is the dilution factor of the standard curve, F is the folate content of the standard, m is the mass of the sample, and *V*_*2*_ is the volume of the sample. This calculation method ensures the accuracy and reproducibility of folate content measurements.

### 2.3 **Deter**min**ation and analysis of the content of folate components**

Samples of freeze-dried root powders from Y25 and 968–19 at different storage roots were prepared, with each sample weighing approximately 100 mg. To the powdered samples, 1 mL of extraction buffer (pH 7.0, 50 mM phosphate buffer; 5% sodium ascorbate; 0.2% β-mercaptoethanol, containing 20 ng/mL methotrexate as an internal standard) was added and thoroughly mixed. The mixture was then boiled for 10 min, followed by cooling on ice until reaching room temperature. Subsequently, 20 μL of α-amylase (40 mg/mL) was added and vortex mixed, and the samples were incubated at 37 °C for 4 h. After incubation, the samples were boiled again for 5 min, followed by cooling on ice for 10 min and then centrifuged at 15000*g* for 10 min at 4 °C. The supernatant was transferred to a three kDa ultrafiltration tube (Millipore, Billerica, MA, USA) for sample purification and centrifuged at 15000*g* for 20 min at 4 °C. The resulting supernatant was collected and transferred to vials for subsequent analysis. Quantitative analysis of folate and its metabolites in the samples was carried out using ultra-high-performance liquid chromatography (UHPLC, Agilent 1290) (Agilent Technologies, Santa Clara, USA) coupled with tandem mass spectrometry (MS/MS, Agilent 6495), (Agilent Technologies, Santa Clara, USA).

### 2.4 Transcriptome sequencing

Samples of tubers from two sweet potato varieties, Y25 and 968–19, at the storage roots S1 and S3, were harvested and stored in liquid nitrogen and −80 °C for total RNA extraction. RNA extraction and sequencing was carried out by Metware Biotechnology Co., Ltd. (Metware Biotechnology, Wuhan, China). In brief, the extracted RNA samples met the quality standards and mRNA isolation was performed, followed by reverse transcription to synthesize complementary DNA (cDNA). The cDNA underwent a series of steps, including end repair, poly-A tailing, adapter ligation, purification, PCR amplification, denaturation, and circularization, to complete the library construction. After library construction, initial quantification was performed using Qubit (Thermo Fisher Scientific, Bremen, Germany), and the insert fragments of the library were assessed using Agilent 2100 (Agilent Technologies, Santa Clara, USA) to ensure library quality. Finally, the effective concentration of the library was accurately quantified using q-PCR, confirming that the effective concentration was above 2 nM, thus providing a high-quality RNA library for subsequent high-throughput sequencing analysis.

### 2.5 Quality control and bioinformatics analysis

Using *Taizhong6* (Wu et al., 2018; Yang et al., 2017) as the reference genome, Clean Reads were aligned to it using HISAT2, and subsequently, StringTie was employed to assemble and stitch the reads based on their positional information. The number of reads for each gene was calculated based on the alignment results and the gene positions on the reference genome. Normalization was performed because some gene fragments are relatively large and contain more reads. FPKM (Fragments Per Kilobase of transcript per Million mapped reads) was used as a metric to identify differentially expressed genes (FDR < 0.05 and log_2_|FoldChange| ≥ 1). The selected differentially expressed genes were compared with KEGG, GO, and KOG databases for functional annotation, and differential expression gene enrichment analysis was conducted, resulting in functional annotations and enrichment analysis outcomes for the differentially expressed genes.

### 2.6 qRT-PCR validation

Before further processing, all samples were treated with a solid RNase removal agent (Coolaber, Beijing, China). The stored root samples were then ground into a powder in liquid nitrogen using a mortar and pestle, followed by RNA extraction using RNaiso Plus (Takara Biotechnology, Beijing, China). An ultra-micro volume spectrophotometer (Bio Drop, Cambridge, UK) was employed to select samples based on the A260/A280 ratio (1.8–2.1), and cDNA synthesis was carried out using the Prime Script™ RT reagent kit (with gDNA wiper) (Takara Biotechnology, Beijing, China). The resulting cDNA samples were diluted fivefold and analyzed via qPCR using TB Green Premix Ex Taq™ II (Takara Biotechnology, Beijing, China) on a real-time fluorescence quantitative PCR system (Touch Real-Time PCR Detection System, Bio-Rad CFX96) (Bio-Rad Laboratories, California, USA). The qPCR conditions were set as follows: 95 °C for 30 s; 40 cycles at 95 °C for 5 s; 60 °C for 30 s. A melt curve analysis (60–95 °C; 0.5 °C increments, lasting 5 s each) was performed to confirm specificity. The relative gene expression was calculated using the ∆∆Ct method with Bio-Rad Manager 3.1 (Bio-Rad Laboratories, California, USA), with *IbActin* serving as the normalization control. Primer design was performed using Primer Premier 5.0 (Supplementary Data Table S2).

### 2.7 *IbADCL1* (*G54242*) gene cloning and overexpression vector construction

Total RNA was extracted from the sweet potato tubers using RNAiso Plus (Takara, Beijing, China), and reverse transcription to synthesize the first-strand cDNA. Specific full-length cDNA primers were designed using Primer Premier 5 software with the following sequences: *IbADCL1*-F: TCTTTCCACCTTTCTTCTA, *IbADCL1*-R: AGTTTCAGTTTGCCTTAGCT. The purified PCR products were subjected to sequencing analysis by a commercial service provider (Sangon Biotech, Shanghai, China). Multiple sequence alignment of the encoded protein sequences was performed using the tool available at http://multalin.toulouse.inra.fr/multalin/, and the tertiary structure of the IbADCL1 protein was predicted and analyzed using the online software available at http://swissmodel.expasy.org/. The overexpression vector was constructed by Shanghai Pujie Biotechnology Co., Ltd. (http://pujiebio.com), using *p2301-35SN-GFP* as the backbone. The p2301-derived expression vector was precisely engineered to 13,459 bp through restriction-ligation cloning, incorporating *XbaI* (TCTAGA) and *KpnI* (GGTACC) recognition sites within the optimized multiple cloning site (MCS) for directional insertion (Supplementary Data Fig. S1). Sequence-verified cloning of the 1042 bp target ORF (positions 2348-3390) was achieved, with the final construct retaining the native kanamycin resistance gene (KanR) for transformant selection in *E. coli DH5α*.

### 2.8 Establishment of genetic transformation system of sweet potato hairy roots mediated by *Agrobacterium rhizogenes*

Retrieve the competent *Agrobacterium rhizogenes K599* cells stored at −80 °C and allow them to thaw slightly at room temperature or in the palm of your hand before immediately placing them back on ice. Add 1.0 μL of plasmid DNA to the competent cells, mix rapidly and vigorously at the bottom of the tube or pipette to ensure even distribution, then incubate the mixture on ice for 5 min, in liquid nitrogen for 5 min, in a 37 °C water bath for 5 min, and finally on ice again for 5 min. Subsequently, add 700 μL of TY liquid medium without antibiotics and incubate the mixture at 28 °C with shaking for 2 h. After that, collect the cells by centrifuging at 6000 rpm for 1 min, discard 700 μL of the supernatant, and evenly spread the remainder on a TY solid medium containing streptomycin (strep). Place the plates upside down in a 28 °C incubator in the dark for 2–3 days.

Single colonies of positive clones were picked and inoculated into 10 mL of TY liquid medium containing kanamycin (Kan, 50 mg·L^−1^) and streptomycin (strep, 250 mg·L^−1^), followed by overnight incubation at 28 °C and 200 rpm. The overnight culture was then diluted 1:1000 in fresh medium and grown at 28 °C and 200 rpm until reaching an OD600 of 1, which was used as the infection liquid. Concurrently, 800 μL of the overnight culture was spread on TY solid medium containing streptomycin and incubated upright at 28 °C for 2 days to serve as the bacterial plate.

Apexes of tissue-cultured sweet potato shoots, maintained under sterile conditions, were used as explants. The apex was cut at a 45° angle approximately 5 cm from the tip, retaining only the terminal leaf. The cut surface was dipped in sufficient microbial colonies from a culture plate before transplanting into nutrient soil. Meanwhile, more than 5 mL of the infection liquid was injected along the root system (the control group received tap water injections after dipping the wound in water. The plants were then placed in a greenhouse to promote tuber growth. After approximately 2 months, the adventitious roots were evaluated, and the positive roots were replanted in the greenhouse for further growth.

### 2.9 Statistical analysis

Data organization and summary were conducted using Microsoft Office Excel 2016. Variance analysis of agronomic traits was performed with SPSS 22.0. Both transcriptomic and metabolomic data were processed and analyzed in depth using the R language (http://www.r-project.org/). All experiments were performed with three biological replicates, each consisting of three technical replicates, to ensure the reliability and accuracy of the results.

## 3 Results

### 3.1 Analysis of folate content in different varieties of sweet potato and screening for representative varieties with different folate content

**Fig. 1:**
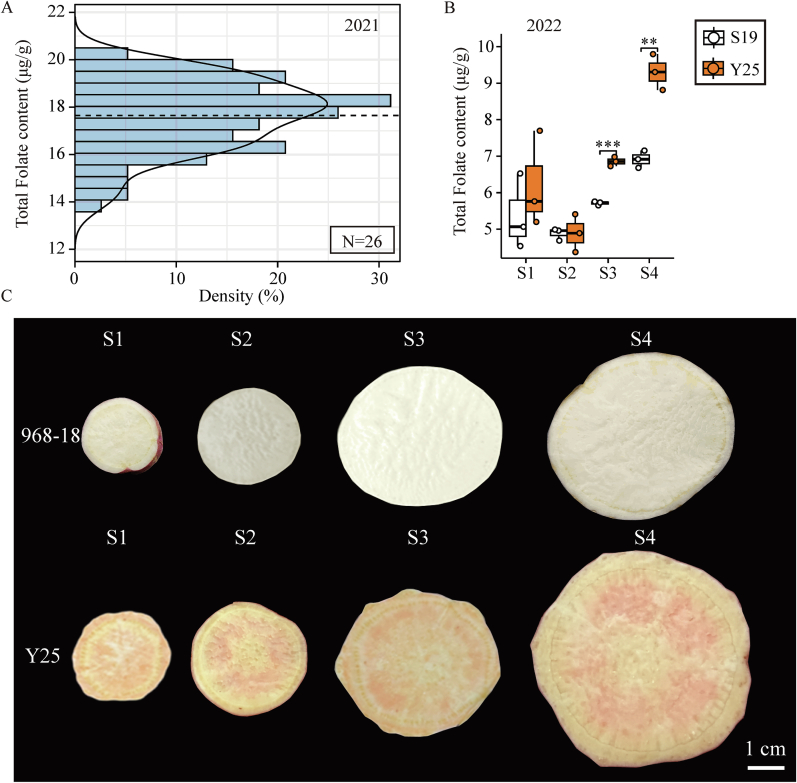
Folate content of sweet potato root tuber and phenotypic map of extreme varieties. A: Total folate content of 26 sweet potato resources in 2021. B: Total folate content of 968–19 and Y25 during storage root expansion stage in 2022. C: Phenotypic map of root tuber of 968–19 and Y25. Data are means ± SEM (*n* = 3). *** *p* < 0.001, ** *p* < 0.01, * *p* < 0.05.

The synthesis and metabolism of folate are intricately regulated by a complex interplay of environmental and genetic factors. To elucidate the molecular mechanisms governing folate synthesis and accumulation during sweet potato storage roots development, it is imperative to mitigate the influence of environmental factors on folate stability. Accordingly, this study assessed the folate content in the storage roots of 26 representative sweet potato varieties. The results indicated that the average folate content in the roots of sweet potato varieties planted in 2021 was 17.71 μg/g. Among these, the Y25 variety exhibited the highest folate content, reaching 19.94 μg/g. In comparison, the 968–19 variety had the lowest folate content at only 14.10 μg/g (Fig. 1A). Based on this screening, two varieties with significant differences in folate content, Y25 and 968–19, were selected for pot-based cultivation under isolated conditions experiments. As shown in Fig. 1B, following pot-based cultivation under isolated conditions, the folate content in both varieties significantly decreased; however, the trend of their differences remained apparent. During the root swelling period, Y25 and 968–19 exhibited a trend of initial decline followed by an increase in folate content, indicating a consistent synthesis and metabolism of folate in sweet potato roots across different developmental stages. During the S1 and S2 stages, the folate content in Y25 was similar to that in 968–19. However, Y25 showed a significantly higher folate content than 968–19 during the S3 and S4 stages. This indicates that folate accumulation in sweet potato roots mainly takes place during the dry matter accumulation phase and increases alongside the accumulation of dry matter.

### 3.2 Folate targeted metabolism analysis of Y25 and 968–19 root tubers

**Fig. 2:**
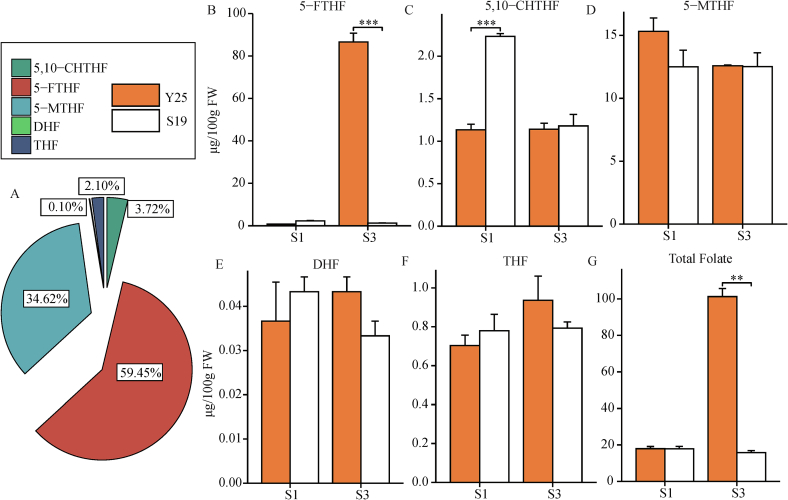
Comparative analysis of total folate and its components in storage root expansion stage of Y25 and 968–19. A: Proportion of each component of folate. B-F: The content changes of Y25 and 968–19 folate components in S1 and S3 periods. G: The content changes of total folate in Y25 and 968–19 at S1 and S3 stages. Data are means ± SEM (*n* = 3). *** *p* < 0.001, ** *p* < 0.01, * *p* < 0.05.

During the formation and development of sweet potato storage roots, the content of various components of folate displays significant dynamic changes. This study aims to elucidate the patterns of folate variation in sweet potato roots by employing targeted metabolomic analysis on root samples from two varieties, Y25 and 968–19, at two key developmental stages, S1 and S3 (Fig. 1C). The analysis revealed five primary folate components: 5,10-methenyl tetrahydrofolate (5,10-CHTHF), 5-formyl tetrahydrofolate (5-FTHF), 5-methyltetrahydrofolate (5-MTHF), dihydrofolate (DHF), and tetrahydrofolate (THF) (Fig. 2A). At the S1 stage, the 5-FTHF content was 0.74 μg/100 g for Y25 and 2.32 μg/100 g for 968–19. As the roots matured, the 5-FTHF content in Y25 significantly increased to 86.58 μg/100 g at the S3 stage, representing a 116-fold increase, while 968–19 exhibited the opposite trend, with 5-FTHF content decreasing to 1.24 μg/100 g, a decline of 1.8 times (Fig. 2B). Furthermore, during the S1 stage, the 5,10-CHTHF content in the 968–19 variety was significantly higher than that in the Y25 variety. However, by the S3 stage, the difference in 5,10-CHTHF content between the two varieties was no longer significant (Fig. 2C). Notably, Y25 showed slightly higher 5-MTHF levels than 968–19 at the S1 stage, but their concentrations converged by the S3 stage (Fig. 2D). Other folate components were present at relatively low levels and showed slight variation between the two developmental stages (Fig. 2E, F). Importantly, while total folate content showed no significant difference between the two varieties at S1, Y25 accumulated dramatically higher total folate than 968–19 at the S3 stage (Fig. 2G). This stark contrast in variation patterns may reflect differences in nutrient allocation and metabolic regulation strategies during root development between the two varieties.

### 3.3 Transcriptome analysis of Y25 and 968–19 sweet potato storage roots

To elucidate the molecular mechanisms underlying folate biosynthesis in sweet potato tubers, we conducted a transcriptomic analysis of selected samples from a metabolomics study, namely tuber samples from two varieties (Y25 and 968–19) at two critical developmental stages (S1 and S3). Using FDR < 0.05 and log2|FoldChange| ≥ 1 criteria, we identified differentially expressed genes (DEGs) in the tubers of Y25 and 968–19 across different developmental stages. Correlation analysis and principal component analysis (PCA) indicated good reproducibility among samples, with significant differences observed between groups (Supplementary Data Fig. S2A, B).

In the comparison between Y25-S1 and 968–19-S1, 5038 DEGs were detected, with 2270 upregulated and 2768 downregulated. In comparing Y25-S3 and 968–19-S3, 7789 DEGs were identified, with 3784 upregulated and 4005 downregulated (Supplementary Data Fig. S2C). This highlights an increasing richness in gene expression differences between the two varieties as sweet potato tubers develop, laying the groundwork for the selection of candidate genes.

In the comparison between 968 and 19-S1 and 968–19-S3, 5288 DEGs were detected, with 2142 upregulated and 3146 downregulated. For the comparison of Y25-S1 and Y25-S3, 5117 DEGs were identified, comprising 2195 upregulated and 2922 downregulated genes (Supplementary Data Fig. S2C). The similarity in the quantity of DEG across developmental stages corroborates the consistency in the developmental processes of the two varieties and aids in minimizing background noise that may affect the results.

By comparing differentially expressed genes (DEGs) with known GO terms, we can identify function categories that are significantly enriched under specific conditions. In this study, GO enrichment analysis was conducted on the selected DEGs (Supplementary Data Table S3-S6). The results revealed that in the comparison between Y25-S1 and 968–19-S1, 889 molecular functions (MF), 2469 biological processes (BP), and 378 cellular components (CC) were enriched. In the Y25-S3 versus 968–19-S3 comparison, 970 MF, 2693 BP, and 439 CC were enriched. The 968–19-S1 versus 968–19-S3 analysis yielded 917 MF, 2555 BP, and 386 CC, whereas the comparison of Y25-S1 and Y25-S3 identified 874 MF, 2487 BP, and 346 CC.

### 3.4 Identification of candidate genes for folate biosynthesis metabolism in sweet potato tubers

**Fig. 3:**
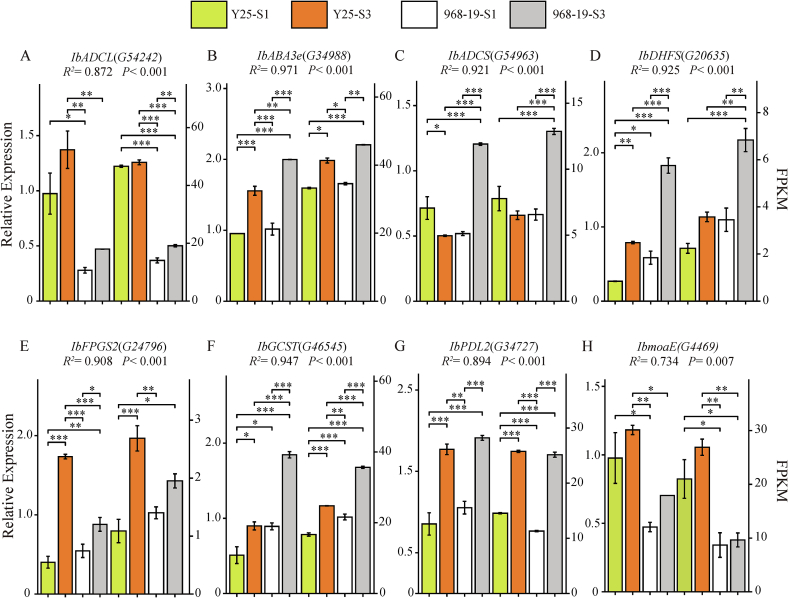
Expression patterns of candidate genes related to folate biosynthesis pathway in sweet potato. Data are means ± SEM (*n* = 3). *** *p* < 0.001, ** *p* < 0.01, * *p* < 0.05.

Folate biosynthesis involves a complex network of metabolic pathways and the regulation of key enzymes (Scott, Rébeillé, & Fletcher, 2000). This study focuses on the mapped pathways directly related to folate biosynthesis. Analyzing the differentially expressed genes (DEGs) within these pathways gives us insights into the key steps and regulatory nodes involved in folate biosynthesis. 31 DEGs were annotated in the folate biosynthesis pathway (ko00790). To visualize their dynamic expression profiles, a heatmap of folate biosynthesis genes (ko00790) across developmental stages was constructed (Supplementary Data Fig. S3). We selected DEGs with higher FPKM values and significant differences for validation against transcriptomic data. The results demonstrated that the expression trends observed through qRT-PCR were consistent with those obtained from RNA-Seq, confirming the reliability of the transcriptomic data (Fig. 3). Subsequently, we screened for candidate genes using the criteria of FPKM >10 and significant differences across all varieties at all time points, ultimately identifying two genes: *G54242* and *G4469*.

*G54242* encodes the enzyme 4-amino-4-deoxychorismate lyase (ADCL), a PLP-dependent lyase that plays a central role in folate biosynthesis. Specifically, ADCL catalyzes the conversion of 4-amino-4-deoxychorismate (ADC) to p-aminobenzoate and pyruvate. This reaction is a key step in folate biosynthesis and is crucial for the growth and development of various organisms. *G4469* serves as the large subunit of molybdopterin (MPT) synthase, playing a critical role in the synthesis of the molybdenum cofactor (Moco), which is essential for the catalytic activity of molybdenum enzymes involved in various biological functions, such as nitrogen, sulfur, and carbon metabolism. *G4469* forms a heterotetrameric enzyme with MoaD, responsible for converting cyclic pyranopterin monophosphate (cPMP) into MPT. Thus, *G54242* was selected as a candidate gene for validating the folate biosynthesis metabolism in sweet potato tubers.

### 3.5 Identification and functional analysis of the *G54242* (*IbADCL1*) gene

**Fig. 4:**
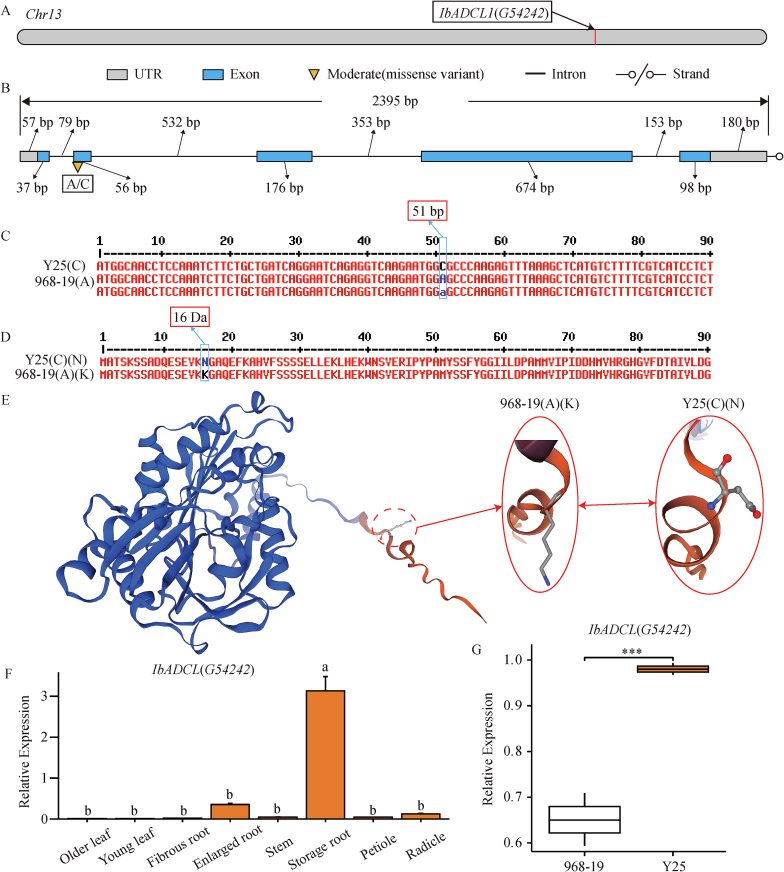
Functional identification of *G54242* (*IbADCL1*). A: The location of *IbADCL1* on chromosome. B: Gene structure of *IbADCL1*. C and D: Gene sequence and amino acid sequence alignment of mutation sites. E: Difference map of protein tertiary structure of mutation sites. F: *IbADCL1* tissue-specific expression (Y25, S4 stage). G: The expression difference of *IbADCL1* in Y25 and 968–19 storage roots (S4 stage). Data are means ± SEM (*n* = 3). *** *p* < 0.001, ** *p* < 0.01, * *p* < 0.05.

*G54242* is located on chromosome 13, with a full gene length of 2395 bp. Based on its functional annotation, this gene has been designated as *IbADCL1*. To investigate potential sequence variations, we cloned the maximum open reading frame (ORF) of this gene and compared the amino acid sequences derived from the cDNA of sweet potato varieties Y25 and 968–19 (Fig. 4A, B). The results indicated a single nucleotide mutation at position 51 bp in the coding region, resulting in a nonsynonymous mutation where aspartic acid (N) at a specific site was replaced by lysine (K) (Fig. 4C, D). Subsequently, we predicted the tertiary structure of the IbADCL protein, revealing that 968–19 lacks one helix compared to Y25 (Fig. 4E), which may have implications for the protein's activity or function. To further elucidate the regulatory mechanisms of *ADCL* in folate biosynthesis and degradation during sweet potato growth and development, we selected the high-expressing variety Y25 for tissue-specific analysis, revealing that its expression level in storage roots was significantly higher than that of other tissues (Fig. 4F, G). In pot experiments, the expression level of *IbADCL1* in Y25 was notably higher than in 968–19, reaching 4.65 times and 3.54 times at stages S1 and S3, respectively. Both varieties exhibited gradually increasing *IbADCL1* expression levels during storage root development (Fig. 3A).

### 3.6 Acquisition and identification of transgenic plants

**Fig. 5:**
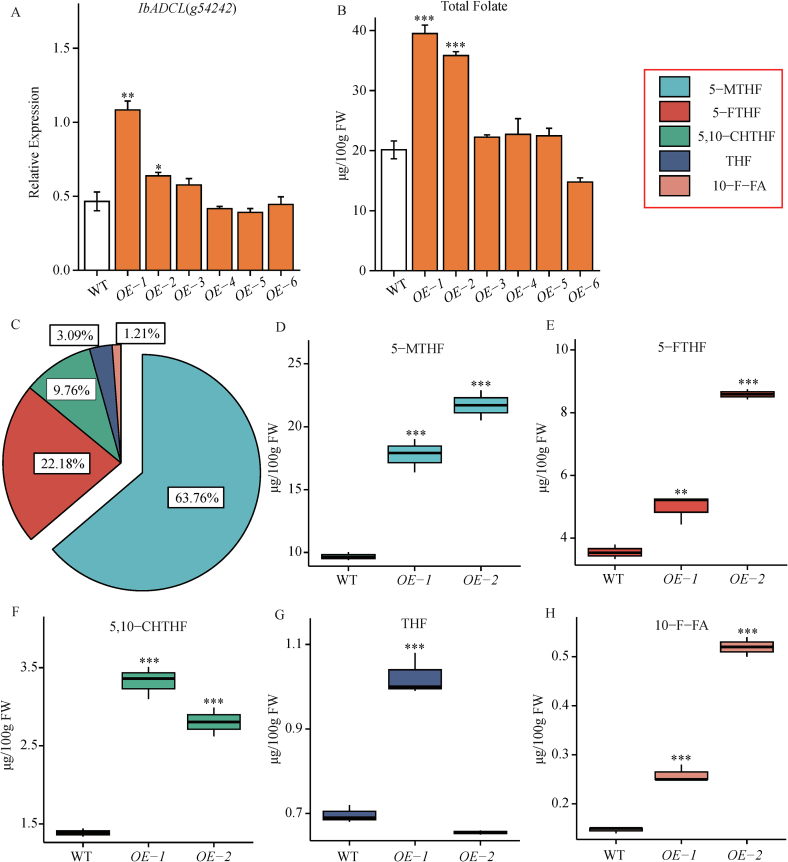
The expression level of transgenic plants and the changes in folate content and composition. A: The expression of IbADCL1 in different overexpression lines. B: The content of total folate in different overexpression lines. C: The proportion of folate components in the overexpressed lines determined by metabolic spectrum. D-H: The content of folate components in WT, *OE-1*, and *OE-2* storage roots. Data are means ± SEM (n = 3). *** p < 0.001, ** p < 0.01, * p < 0.05.

In the past few decades, traditional breeding has played a significant role in improving sweet potato traits. However, due to the complex genetic background of sweet potato, characterized by a high rate of male sterility, self-incompatibility, and interspecific incompatibility, many valuable traits have been lost during this process (Gama, Leite, Cordeiro, & Cantliffe, 1996; Yan et al., 2022). This study draws on the methods of the Cut-Dip-Budding (CDB) genetic transformation system (Cao et al., 2024). It utilizes *Agrobacterium*-mediated transformation to induce transgenic plants from sweet potato adventitious roots. The resulting overexpression *IbADCL* transgenic plants were designated *OE-1*, *OE-2*, *OE-3*, *OE-4*, *OE-5*, and *OE-6*. Using genomic DNA from both the transgenic and wild-type plants as templates, we designed primers to amplify the *GFP* gene sequence for identification. PCR analysis (Supplementary Data Fig. S4) showed that while all six sweet potato plants were confirmed as transgenic lines, the *OE-1* and *OE-2* lines exhibited the most prominent banding patterns. Subsequently, we employed qRT-PCR to detect the relative expression levels of the *IbADCL* gene in the six transgenic sweet potatoes compared to the wild type (Fig. 5A). The results revealed that *OE-1* and *OE-2* exhibited significantly higher relative expression levels than the wild type (WT), successfully achieving overexpression of the *IbADCL* gene. Specifically, the expression levels of the *IbADCL* gene in *OE-1* and *OE-2* were 2.33-fold and 1.37-fold higher than that of the wild type (WT), respectively. We then measured the total folate content in the tubers of these six sweet potato lines, and the results indicated that the total folate content in *OE-1*, *OE-2*, and *OE-4* was significantly increased (Fig. 5B), with increases of 96%, 78%, and 79%, respectively. Therefore, we selected *OE-1* and *OE-2* for subsequent targeted folate metabolomics analysis.

Through targeted folate metabolomics analysis of the overexpressing plants, we aimed to visually demonstrate the impact of the *IbADCL1* gene on folate biosynthesis in sweet potato tubers. The total folate content in the wild-type (WT) sweet potato was 15.45 μg/100 g. Both overexpression lines, *OE-1* and *OE-2*, exhibited a significant increase in total folate content, with increases of 77% and 122%, respectively. *OE-2* showed the highest total folate content, reaching 34.27 μg/100 g. Furthermore, following the overexpression of the *IbADCL1* gene, 5-MTHF and 5-FTHF remained the predominant forms of folate in the sweet potato tubers, with significantly increased levels of these two components. Notably, the concentration of 5-MTHF increased by 184% to 224% compared to that of WT. The level of 5-FTHF also increased significantly, with *OE-1* and *OE-2* showing increases of 40% and 142% compared to WT. In *OE-1*, the levels of 5–10-CHTHF and THF were higher than those in *OE-2*, whereas the THF level in *OE-2* was comparable to that in WT. Although 10-F-FA had the lowest concentration among all components, it still exhibited a significantly higher level in the overexpressing lines than in WT (Fig. 5C-H).

## 4 Discussion

The findings of this study provide critical insights into the molecular and metabolic mechanisms underlying folate biosynthesis and accumulation in sweet potato storage roots, highlighting the interplay between genetic factors and developmental regulation. By integrating targeted metabolomics, transcriptomics, and functional genetic validation, this work advances our understanding of folate dynamics in sweet potatoes and offers a foundation for biofortification strategies to enhance nutritional quality.

### 4.1 Genetic and developmental regulation of folate accumulation

The significant variation in folate content among 26 sweet potato varieties underscores the genetic basis of folate biosynthesis. The selection of Y25 (high folate) and 968–19 (low folate) for further analysis revealed that folate accumulation is developmentally regulated, peaking during the dry matter accumulation phase (S3–S4). This aligns with studies in other crops, where folate synthesis is tightly linked to sink tissue development and resource allocation (Farré et al., 2011). The observed decline in folate content under pot-based cultivation under isolated conditions, despite retained differences between varieties, suggests that environmental factors (e.g., soil nutrients, microclimate) modulate folate levels, but genetic determinants dominate. The dynamic changes in folate components (e.g., 5-FTHF and 5-MTHF) during root swelling further emphasize stage-specific metabolic shifts, likely driven by transcriptional reprogramming.

### 4.2 Metabolic shifts and transcriptional drivers

Targeted metabolomics revealed stark contrasts between Y25 and 968–19 in 5-FTHF dynamics, with Y25 exhibiting a 116-fold increase at S3 versus a decline in 968–19. This divergence points to varietal differences in folate salvage pathways or regulatory feedback mechanisms. For instance, the surge in 5-FTHF in Y25 may reflect enhanced activity of folate biosynthesis enzymes (e.g., ADCL) or reduced turnover, while 968–19's decline could indicate prioritization of other metabolic sinks.

The substantial increase of 5-FTHF in Y25 may be linked to the high demand for folate during the maturation stage, involving crucial physiological processes such as DNA synthesis and repair, thus supporting rapid root growth and development. Conversely, the decrease in 5-FTHF content in 968–19 may indicate higher utilization efficiency of this compound during maturation, or alternative metabolic pathways may convert it into other forms of folate or engage it in other metabolic reactions, resulting in the observed reduction. As a one‑carbon substituted derivative of tetrahydrofolate, 5-FTHF serves as an intracellular storage form of folate in folate-mediated one‑carbon metabolism and can inhibit the activity of folate-dependent enzymes such as amino deoxyuridine-5′-monophosphate formyltransferase (AICARFT) and serine hydroxymethyltransferase (SHMT) (Liu et al., 2022; Misselbeck, Marchetti, Priami, Stover, & Field, 2019) The significant increase in 5-FTHF content in the Y25 variety at maturity may confer an advantage in coping with environmental stress and maintaining metabolic stability within cells.

In addition to 5-FTHF, we observed the Y25 variety exhibited a slight decrease in 5-MTHF content from the S1 to S3 stages, while the 968–19 variety showed virtually no change between these two stages (Fig. 2D). 5-MTHF is an active form of folate that plays a crucial role in methyl transfer reactions, particularly in the remethylation of homocysteine to methionine and other important physiological processes. The decrease in 5-MTHF content in the Y25 variety may be associated with changes in its methyl requirements during storage root development (Zierer, Rüscher, Sonnewald, & Sonnewald, 2021). Specifically, during the S1 stage, higher methylation levels may be necessary to support early growth and metabolic regulation. As development progresses to the S3 stage, the demand for methylation decreases, leading to a slight reduction in 5-MTHF levels. In contrast, the stability of 5-MTHF content in the 968–19 variety may suggest a more balanced requirement for methylation across different developmental stages. This stability could also indicate other metabolic mechanisms that help maintain relatively constant levels of 5-MTHF.

Transcriptomic analysis further identified thousands of DEGs between varieties and developmental stages, with enriched GO terms related to folate metabolism, cofactor biosynthesis, and stress responses. The increasing richness of DEGs at later stages (e.g., 7789 DEGs at S3) underscores the complexity of transcriptional networks governing folate accumulation during tuber maturation.

The enrichment results for molecular function (MF) indicate significant differences in the catalytic and transporter activities of the DEGs, likely related to the expression changes of key enzymes and transport proteins in the folate biosynthesis pathway. The biological process (BP) enrichment results further elucidate the involvement of these genes in biological processes such as DNA repair and signal transduction, suggesting their potential roles in folate synthesis and metabolic regulation. The cellular component (CC) enrichment results reveal differences in the localization of DEGs within cellular structures such as the nucleus and mitochondria, which may be associated with the functions of organelles and metabolic activities related to folate synthesis.

By comparing differentially expressed genes (DEGs) with known KEGG pathways, we can identify metabolic pathways that are significantly enriched under specific conditions. KEGG pathway analysis revealed DEG enrichment in multiple metabolic pathways, including Metabolic pathways, Cysteine and methionine metabolism, Biosynthesis of amino acids, Carotenoid biosynthesis, Biosynthesis of secondary metabolites, Starch and sucrose metabolism, Plant hormone signal transduction, and Glutathione metabolism (Supplementary Data Fig. S5-S8). These pathways are closely related to the biosynthesis and metabolic regulation of folate.

The enrichment analysis of cysteine and methionine metabolism pathways indicates that differentially expressed genes participate in sulfur amino acid metabolism, which may be closely related to the expression changes of key enzymes and metabolites in folate biosynthesis. For instance, the enzyme MAT2A in the methionine cycle plays a crucial role in regulating intracellular methylation reactions, and its activity fluctuations may affect folate metabolism. Additionally, the enrichment results from the glutathione metabolism pathway reveal the involvement of differentially expressed genes in the antioxidant defence system. As the primary antioxidant within cells, alterations in glutathione metabolism could impact the stability and bioavailability of folate. The enrichment analysis of starch and sucrose metabolism pathways indicates the role of differentially expressed genes in carbohydrate metabolism, which may relate to the role of folate in energy metabolism. Lastly, the enrichment results from the plant hormone signal transduction pathway demonstrate the involvement of differentially expressed genes in plant hormone signaling. The enrichment of auxin signaling genes specifically suggests crosstalk between folate metabolism and root development, potentially mediated by one‑carbon units serving as precursors for hormone synthesis. Hormones such as auxin and gibberellin play critical roles in regulating plant growth and development, and changes in these signaling pathways may subsequently influence the biosynthesis and accumulation of folate.

### 4.3 *IbADCL1* as a key regulator of folate biosynthesis

The identification of *IbADCL1* (*G54242*) as a candidate gene highlights its pivotal role in folate biosynthesis. ADCL catalyzes the conversion of ADC to p-aminobenzoate, a rate-limiting step in folate synthesis. The biosynthesis pathway of folate in plants and bacteria consists of two main steps: first, the conversion of branch chain acids into 4-amino-4-deoxychorismic acid (ADC), which is catalyzed in plants by the enzyme 4-amino-4-deoxychorismic acid synthase (ADCS), while in bacteria, this process is facilitated by two heterodimeric proteins, PabA and PabB (McIntosh, Brushett, & Henry, 2008). Studies indicate that the ADCS enzyme itself lacks lyase activity, suggesting the presence of other enzymes capable of catalyzing the cleavage of ADC in plants. This hypothesis was validated in *Escherichia coli* with the identification of 4-amino-4-deoxychorismic acid lyase (ADCL), which exhibits dimeric activity (Mehrshahi et al., 2010). Overexpression of *AtADCS* in rice has been shown to significantly increase grain ρ-ABA content, while simultaneously causing a tenfold decrease in folate concentration (Storozhenko et al., 2005). Further studies have demonstrated that folate levels can only be elevated when both *ADCS* and *GCH* are overexpressed together, resulting in a 100-fold increase in folate content in rice grains (Liang et al., 2019). In Arabidopsis, ectopic overexpression of *SiADCL1* increased folate content by 1.14 to 1.84 times (Hou et al., 2022). As a model crop for studying folate, Tomatoes showed that the activity of ADCL was unresponsive to high concentrations of both ρ-ABA and folate, resulting in unaltered expression levels (Mehrshahi et al., 2010). Research by Basset et al. (Basset et al., 2004) revealed that gene expression in the early steps of folate biosynthesis in tomato gradually decreased during development. However, in fruits engineered to overexpress the exogenous *ADCS* gene, the expression of endogenous genes remained unchanged, while *ADCL* expression increased by 7.8 times. Robinson et al. (Robinson et al., 2019) proposed that the expression of the *ADCL* gene is positively correlated with ρ-ABA levels, thereby promoting folate synthesis. Hou et al. (Hou et al., 2022) conducted a combined analysis of folate metabolites and related gene expression levels in six millet varieties, finding a significant positive correlation between *SiADCL1* and total folate content. Therefore, it can be concluded that the high expression of *IbADCL1* may be closely associated with folate accumulation.

The nonsynonymous mutation (N → K) in 968–19, accompanied by a structural truncation (loss of a helix), likely impairs enzyme activity, explaining its lower folate content. Transgenic overexpression of *IbADCL1* in sweet potato significantly increased total folate (up to 122% in *OE-2*), particularly 5-MTHF and 5-FTHF, confirming its functional role. These results mirror findings in Arabidopsis and rice, where *ADCL* overexpression enhanced folate levels, validating its conserved role across species.

**Fig. 6:**
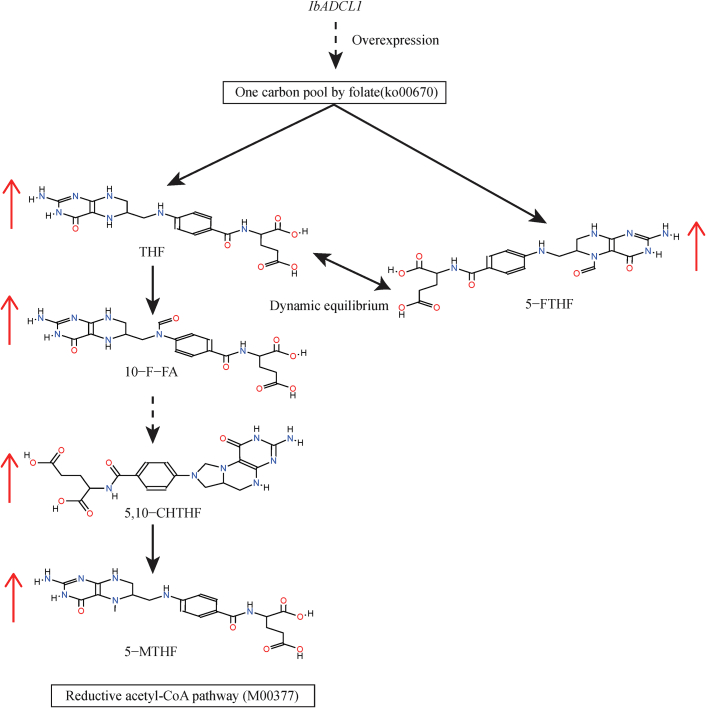
The regulation mechanism of *IbADCL1* gene on folate.

Importantly, no significant changes were observed in other traits, indicating that the overexpression of the *IbADCL1* gene can significantly enhance the biosynthetic capacity of folate in sweet potato tubers without affecting other functions. Although the *IbADCL1* gene is classified under the folate biosynthesis pathway (Ko00790), it lies at the interface between the folate biosynthesis pathway and the one carbon pool by the folate pathway. Its overexpression could potentially affect the downstream one carbon pool by folate pathway. In this study, targeted folate metabolite analysis were mapped onto the KEGG pathways. The results revealed that THF, 10-F-FA, 5,10-CHTHF, and 5-MTHF are all metabolites within the reductive acetyl-CoA pathway (M00377) module. As a key module of the one‑carbon pool by folate pathway, the collective increase in the metabolite contents of M00377 further supports the impact of *IbADCL1* on the one‑carbon pool by folate (Fig. 6). Additionally, 5-FTHF, a derivative of THF, is also located in the one carbon pool by folate pathway and can easily convert to THF without the action of dihydrofolate reductase (DHFR) (Scaglione & Panzavolta, 2014). The increase in its content in overexpressing plants further confirms the critical role of the *IbADCL1* gene in folate biosynthesis. These findings provide robust evidence for enhancing folate content in sweet potato storage roots through genetic engineering approaches.

### 4.4 Implications for biofortification and breeding

The success of the CDB transformation system in generating high-folate lines (*OE-1*, *OE-2*) demonstrates the potential of genetic engineering to augment nutritional traits in sweet potato, a crop plagued by breeding challenges due to genetic complexity and reproductive barriers. The significant increase in 5-MTHF (a bioactive folate form critical for human health) further underscores the translational value of this work. However, the variability in folate components among transgenic lines (e.g., THF levels in *OE-2* vs. *OE-1*) suggests additional regulatory layers, such as post-translational modifications or interactions with other Moco-dependent enzymes (e.g., *G4469*-encoded MPT synthase).

### 4.5 Limitations and future directions

While this study focuses on *IbADCL1*, the role of *G4469* (MPT synthase) warrants exploration, as Moco is essential for enzymes like nitrate reductase, which may indirectly influence folate metabolism. Field trials are needed to assess the stability of folate enhancement under diverse agroecological conditions. Furthermore, multi-omics integration (e.g., proteomics, flux analysis) could unravel post-transcriptional regulation and metabolic flux redistribution.

## CRediT authorship contribution statement

**Qingming Ren:** Writing – original draft, Methodology, Investigation, Conceptualization. **Yingzhi Wu:** Writing – original draft, Methodology, Investigation, Conceptualization. **Huiyu Gao:** Visualization, Formal analysis, Data curation. **Qi Ma:** Writing – review & editing, Validation, Resources. **Xinli Liu:** Software, Investigation, Data curation. **Yinghui Li:** Resources, Project administration, Investigation. **Xiaoxi Zhen:** Writing – review & editing, Validation. **Yuanhuai Han:** Writing – review & editing, Supervision, Funding acquisition. **Bin Zhang:** Writing – review & editing, Supervision, Funding acquisition.

## Declaration of competing interest

The authors declare that they have no known competing financial interests or personal relationships that could have appeared to influence the work reported in this paper.

## Data Availability

RNA-Seq data are available in the NCBI SRA under accession PRJNA1221330.

## A. Supplementary data

Supplementary material 1 Appendix Subheadings Supplementary material 1

Supplementary material 2 Appendix Images Supplementary material 2
