# Supplementary material for: Integrated transcriptomic and metabolomic profiling identifies IbADCL1 as a key regulator of folate biosynthesis in sweet potato storage roots
Source: Food Chem (Oxf). 2025 Sep 13;11:100302. doi: 10.1016/j.fochms.2025.100302 (PMC12494576; doi:10.1016/j.fochms.2025.100302)
Supplement: Supplementary material 1 — Appendix Subheadings [file mmc1.docx]

**Fig S1** Transcriptomic quality control analysis of sweet potato root samples

A: Correlation fraction. B: Principal component analysis of each sample. C: Statistical analysis of differential genes in each comparison group.

**Fig. S2** *GFP* positive identification.

**Fig. S3** KEGG classifications between Y25-S1 and 968-19-S1.

**Fig. S4** KEGG classifications between Y25-S3 and 968-19-S3.

**Fig. S5** KEGG classifications between 968-19-S1 and 968-19-S3.

**Fig. S6** KEGG classifications between Y25-S1 and Y25-S3.

**Fig S7** Schematic diagram of the gene overexpression vector

**Table S1** Y25-S1_vs_968-19-S1GO enrichment.

**Table S2** Y25-S3_vs_968-19-S3GO enrichment.

**Table S3** 968-19-S1_vs_968-19-S3GO enrichment.

**Table S4** Y25-S1_vs_Y25-S3GO enrichment.

**Table S5** Sweet potato resource names and abbreviations.

**Table S6** Differential gene expression primer information.
