# Supplementary material for: Integrated transcriptomic and metabolomic profiling identifies IbADCL1 as a key regulator of folate biosynthesis in sweet potato storage roots
Source: Food Chem (Oxf). 2025 Sep 13;11:100302. doi: 10.1016/j.fochms.2025.100302 (PMC12494576; doi:10.1016/j.fochms.2025.100302)
Supplement: Supplementary material 2 — Appendix Images [file mmc2.docx]

***
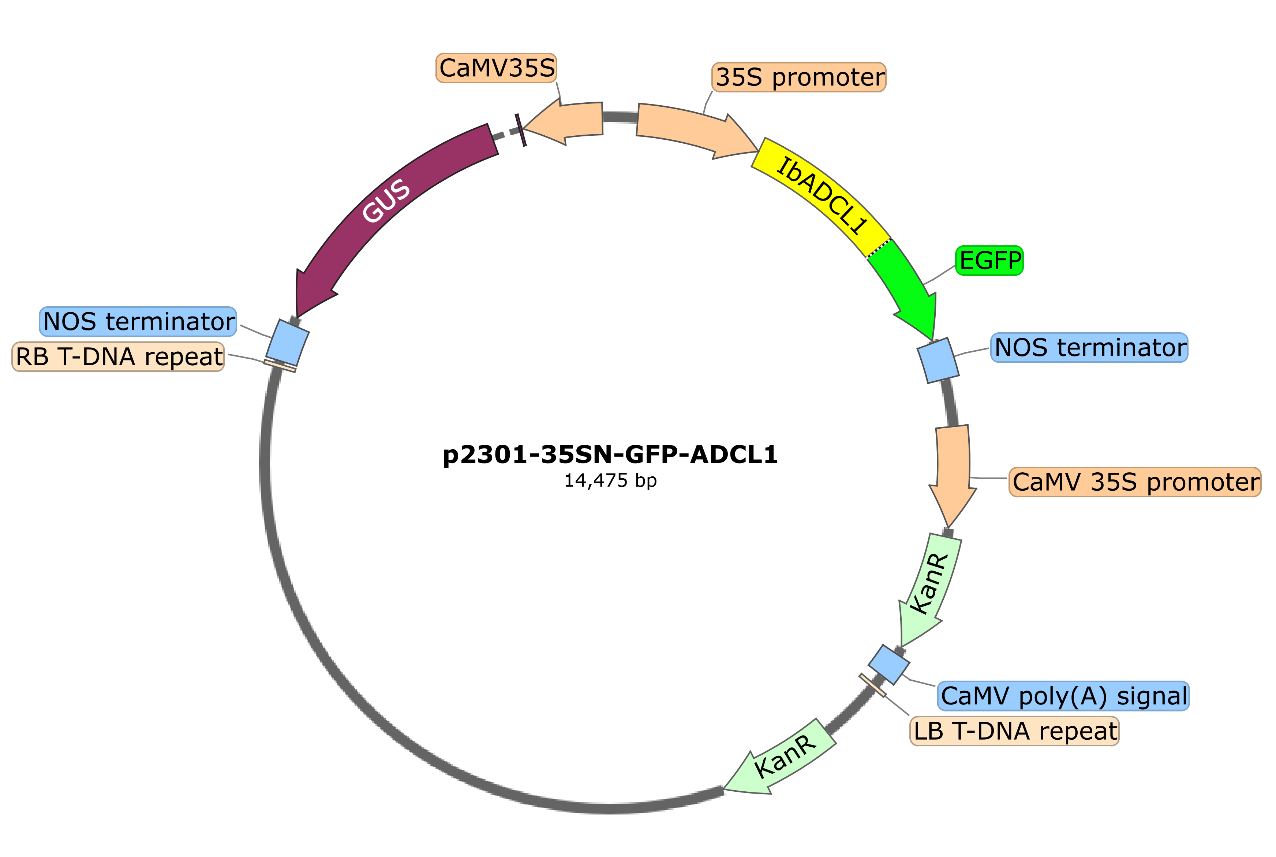
***

**Figure S1** Schematic diagram of the gene overexpression vector


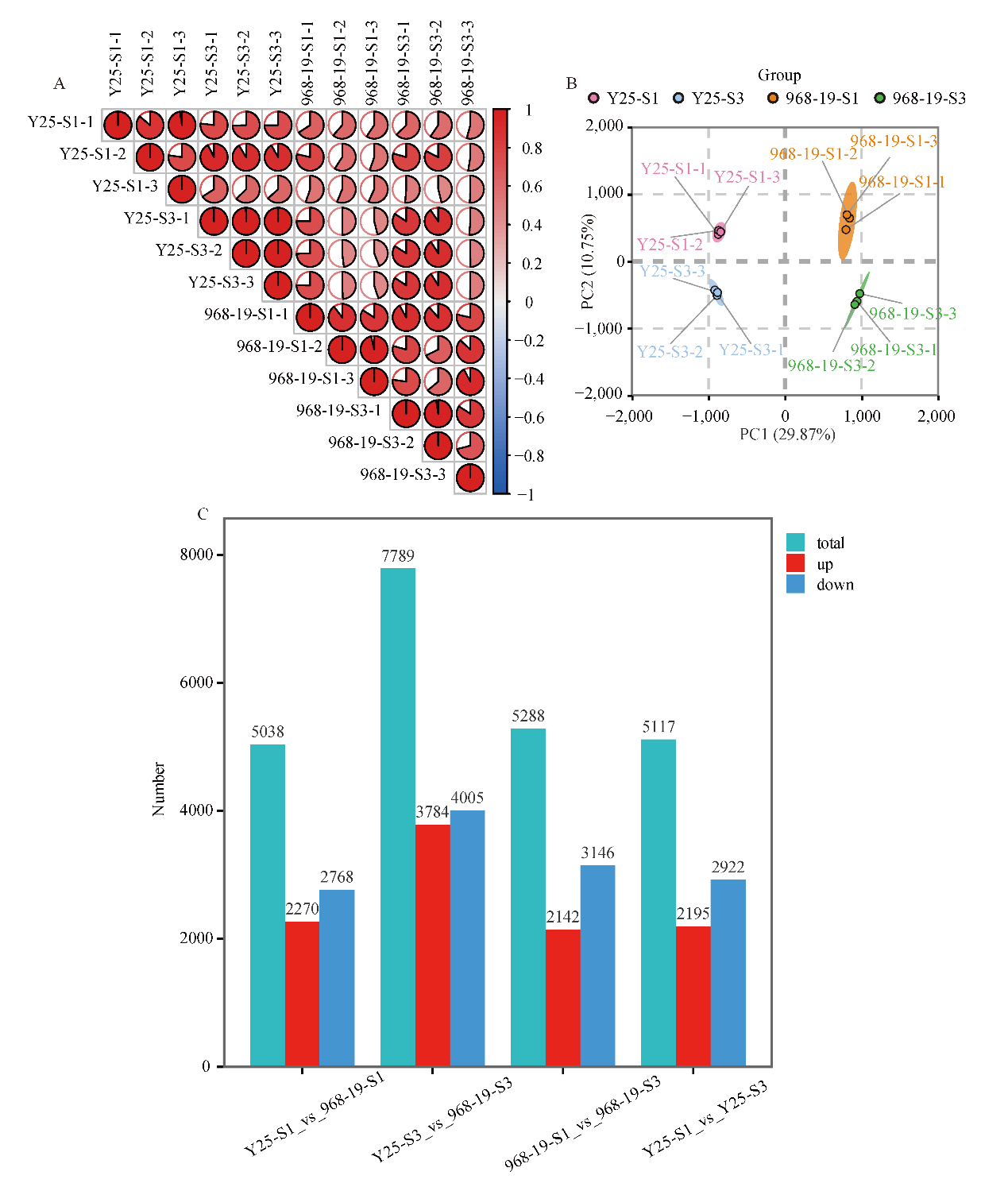


**Figure S2** Transcriptomic quality control analysis of sweet potato root samples

A: Correlation fraction. B: Principal component analysis of each sample. C: Statistical analysis of differential genes in each comparison group.


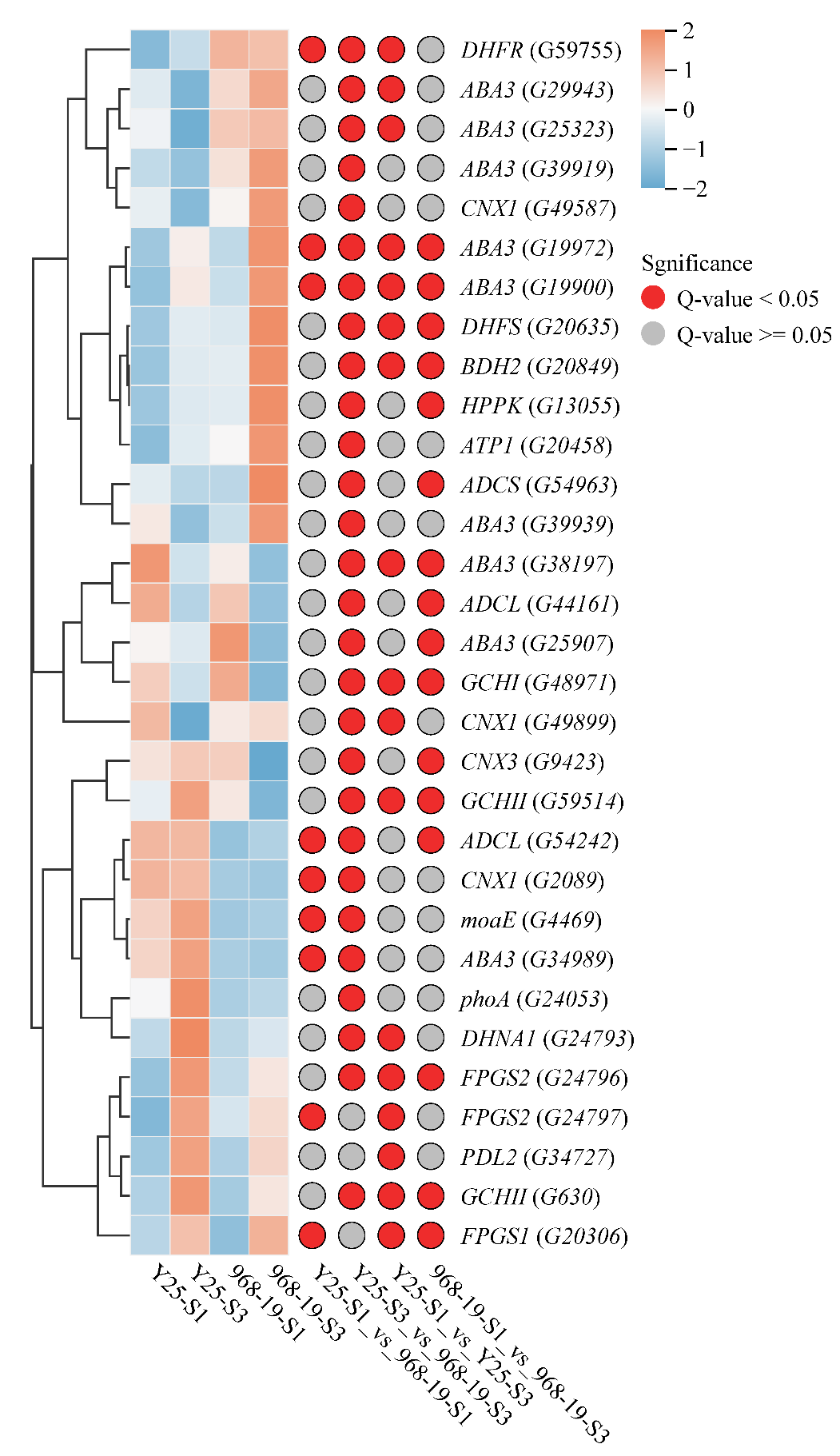


**Figure S3** Folic acid biosynthesis pathway (ko00790) DEGs expression trend heat map

***
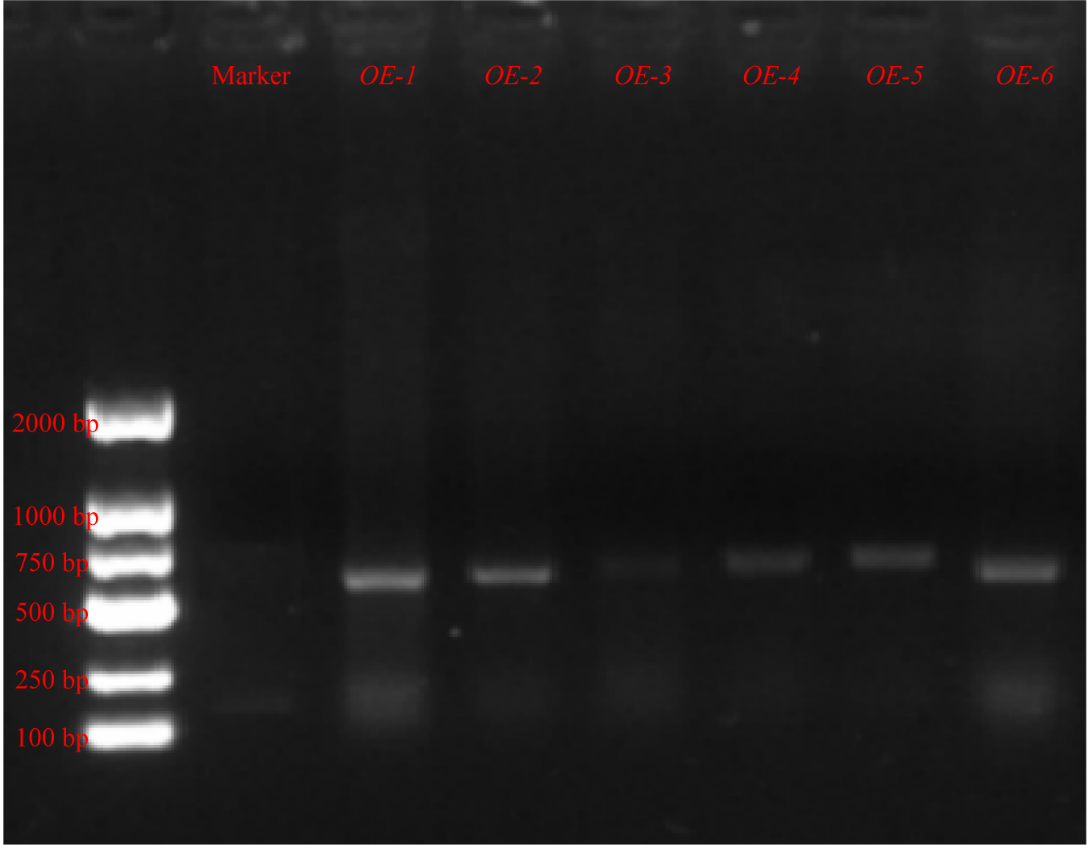
***

**Figure S4** *GFP* positive identification


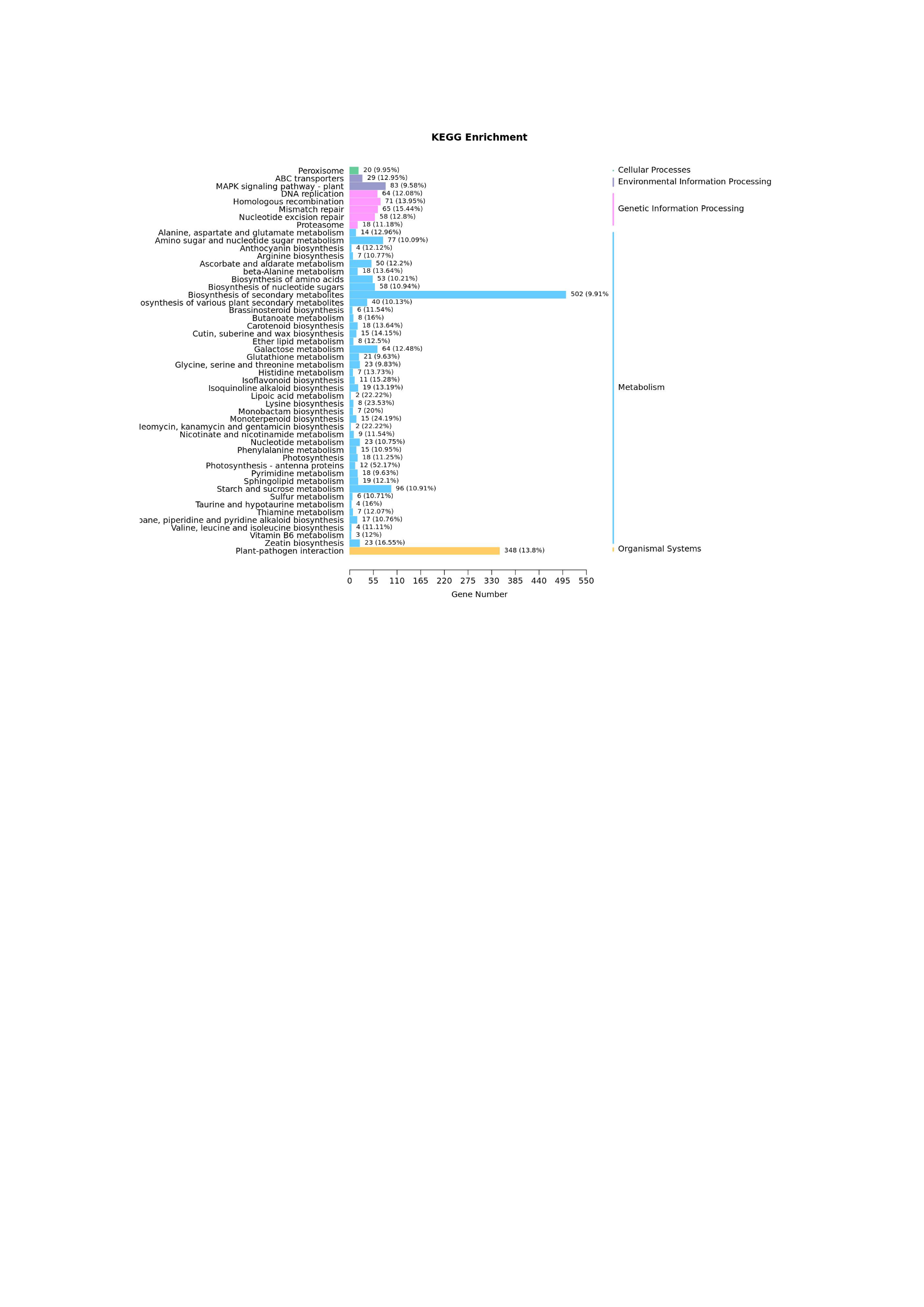

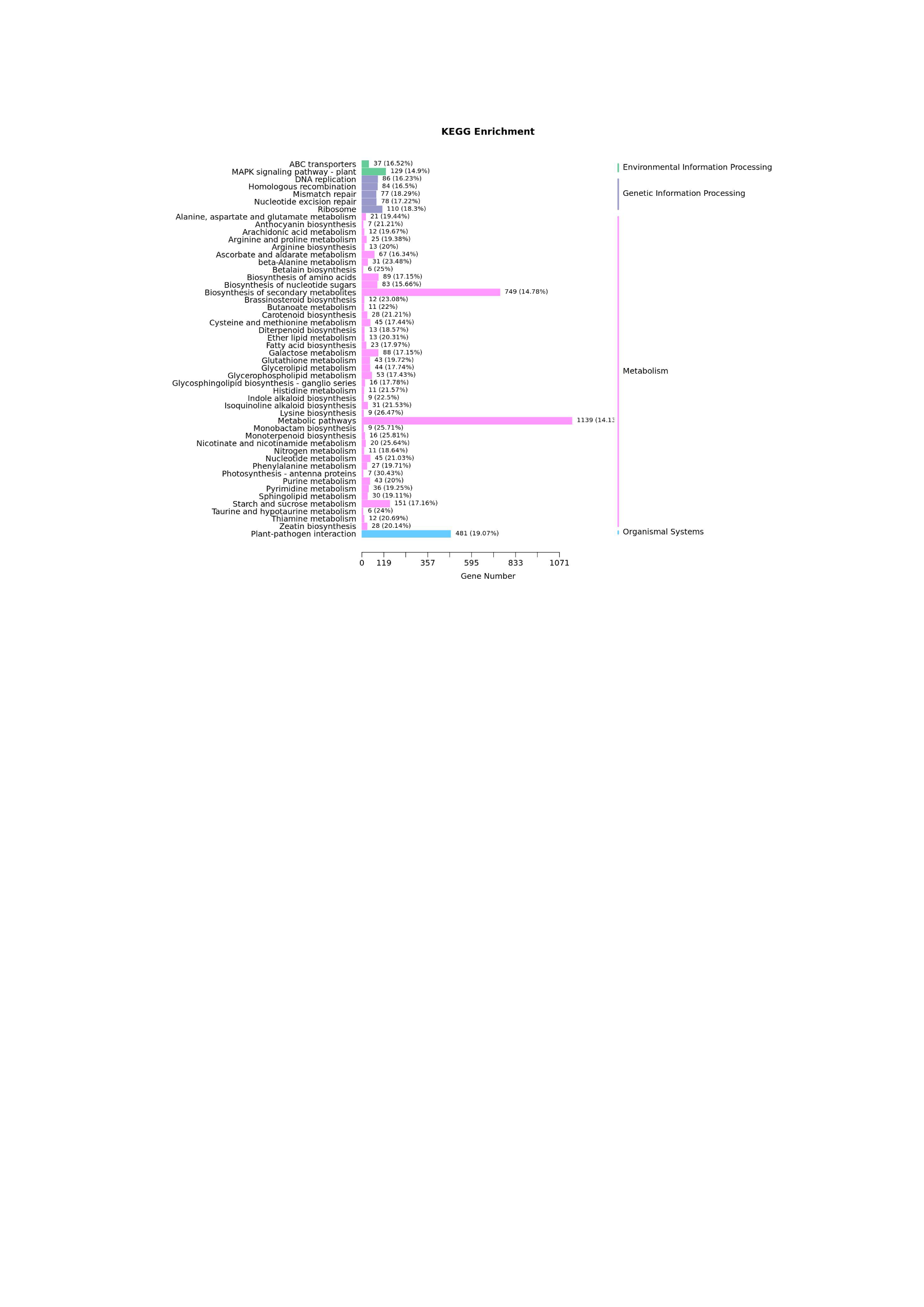
**Figure S5** KEGG classifications between Y25-S1 and 968-19-S1

**Figure S6** KEGG classifications between Y25-S3 and 968-19-S3


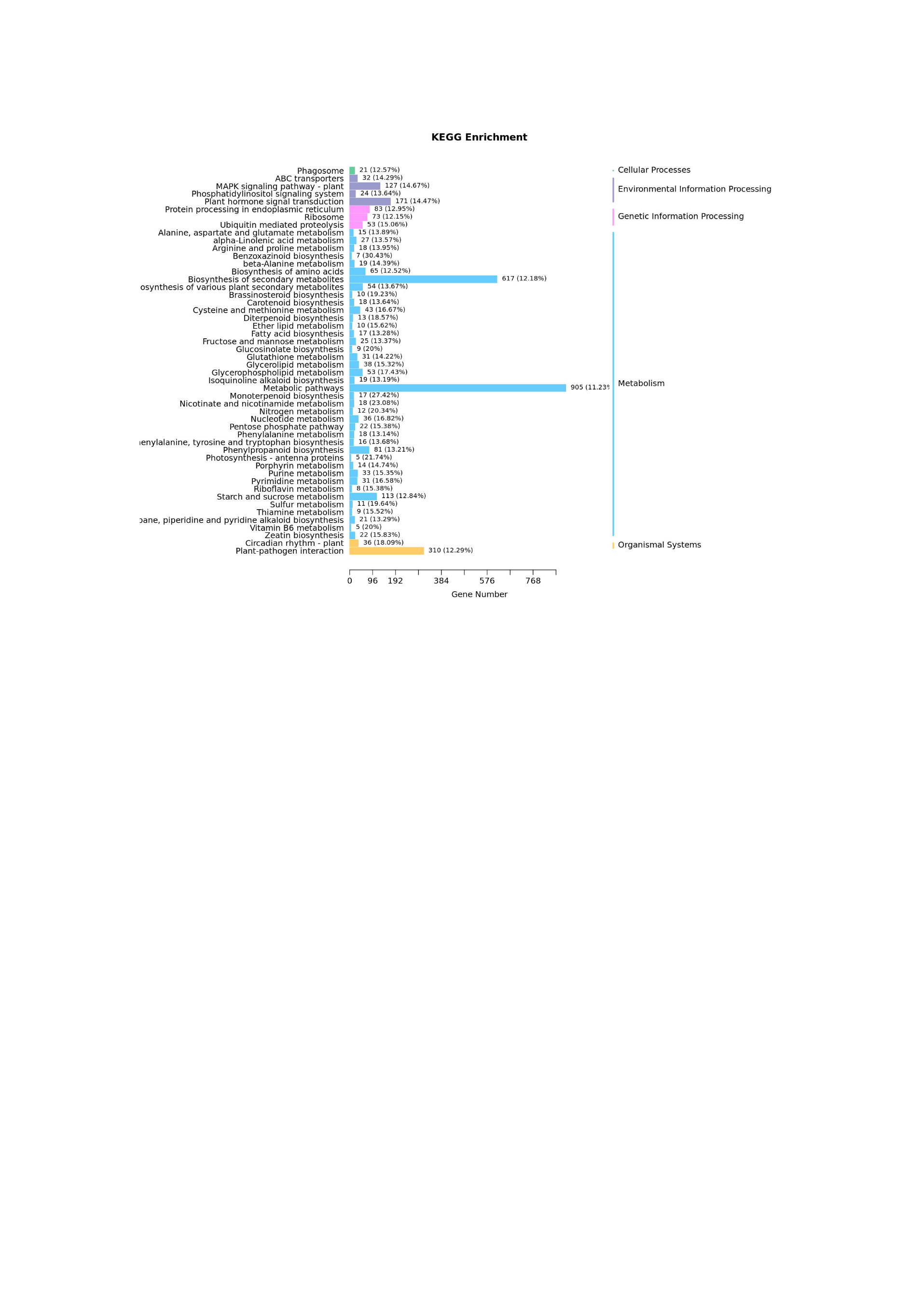

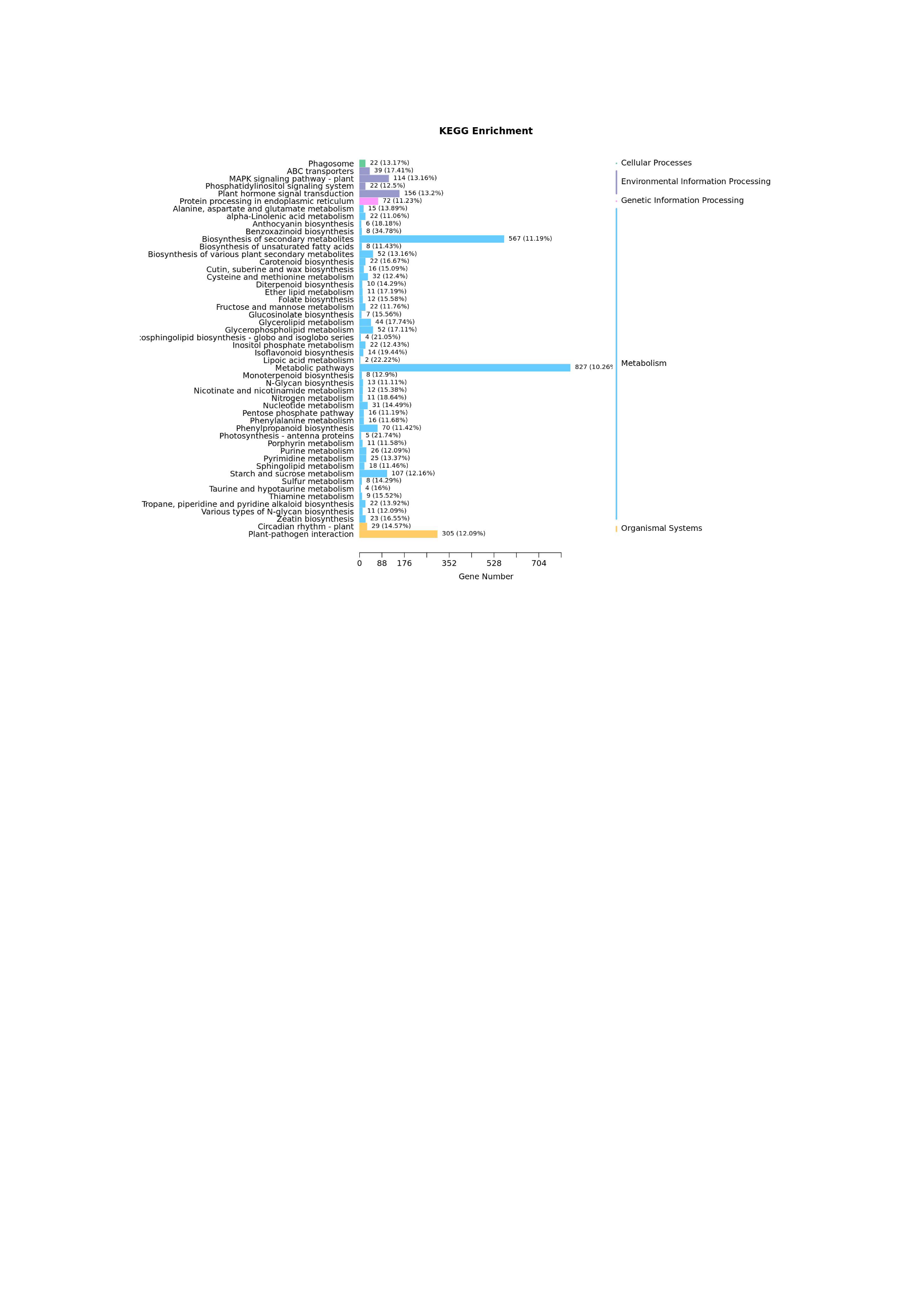
**Figure S7** KEGG classifications between 968-19-S1 and 968-19-S3

**Figure S8** KEGG classifications between Y25-S1 and Y25-S3
