# Supplementary material for: Integrated transcriptomic and metabolomic profiling identifies IbADCL1 as a key regulator of folate biosynthesis in sweet potato storage roots
Source: Food Chem (Oxf). 2025 Sep 13;11:100302. doi: 10.1016/j.fochms.2025.100302 (PMC12494576; doi:10.1016/j.fochms.2025.100302)
Supplement: Supplementary material 3 — Table S1 and S2 [file mmc3.docx]

**Table S1** Name of sweet potato resources and total folic acid content

| Sample name | Abbreviation | Total folic acid content (μg/g) |
| --- | --- | --- |
| Yan Shu 25 | Y25 | 19.94 |
| Hong Dong | HD | 19.88 |
| Xi Meng | XM | 19.82 |
| Bai Shu | BS | 19.46 |
| Xiao Shu | XS | 19.45 |
| Zi Yunhong | ZHY | 19.21 |
| Pu Shu 32 | P32 | 19.12 |
| Liao Niandun | LND | 18.81 |
| Australian Purple and White | OZB | 18.47 |
| Cai Shu | CS | 18.23 |
| Jin Anna | JAN | 18.06 |
| Hong Yao | HY | 18.04 |
| Ha Mi | HM | 18.00 |
| Huang Su 8 | HS8 | 17.92 |
| Xin Xiang | XX | 17.83 |
| Xu Shu 27 | X27 | 17.82 |
| Yi Dianhong | YDH | 17.30 |
| Chun Tian | CT | 16.83 |
| 55-2 | 55-2 | 16.67 |
| Ji Nan 26 | J26 | 16.55 |
| Ming Men Jinshi | MJS | 16.29 |
| Long Shu 9 | L9 | 16.23 |
| Feng Miguan | FMG | 16.14 |
| Qin Shu 5 | Q5 | 15.73 |
| Sheng Li Baihao | SLBH | 14.62 |
| Shang Shu 9 | 968-19 | 14.10 |

**Table S2** Gene primer information

| Gene | Primer(5’-3’) | Amplicon length (bp) |
| --- | --- | --- |
| *G54963* | F: GTGTATGCGAGCCTGGCT | 149 |
|  | R: ACCACCTGGGAATGCAGC |  |
| *G54242* | F: GCGGATGCACTGCTCTGA | 115 |
|  | R: TCAGCAGCTCCTTTGGCC |  |
| *G20635* | F: CGGTTGCTGGAGTGCTCA | 140 |
|  | R: TCCTGCGCCTTTGGGTAC |  |
| *G24796* | F: TGCCTCAACCTTCTGAAGCA | 150 |
|  | R: GGGCAGCATTTGTGAGCT |  |
| *G34988* | F: TTCAGGAGGCAGAGCGTG | 132 |
|  | R: CCCAACCCTCGCAGAAGG |  |
| *G4469* | F: CTGGGACCCGTTGCAGTC | 84 |
|  | R: TTTGCACGCATCCAACGC |  |
| *G34727* | F: CATGCCGTGGGTGGACTT | 89 |
|  | R: ACTTCCCTCCTCAGAAGCTCT |  |
| *G46545* | F: CACCAGCGGAGGGTTCAG | 109 |
|  | R: CTTGACGTTTGTGCCCGC |  |
| *GFP* | F: ATGGTGAGCAAGGGCGAG | 612 |
|  | R: CTCGCCCTTGCTCACCAT |  |
